# Intra-Arterial Radioligand Therapy in Brain Cancer: Bridging Nuclear Medicine and Interventional Neuroradiology

**DOI:** 10.3390/diagnostics16020341

**Published:** 2026-01-21

**Authors:** Federico Sabuzi, Luca Filippi, Mariafrancesca Trulli, Fabio Domenici, Francesco Garaci, Valerio Da Ros

**Affiliations:** 1 Department of Biomedicine and Prevention, University Hospital of Rome “Tor Vergata”, Via Montpellier 1, 00133 Rome, Italy; federico.sabuzi@ptvonline.it (F.S.); mariafrancesca.trulli@ptvonline.it (M.T.); francesco.garaci@uniroma2.it (F.G.); valerio.da.ros@uniroma2.it (V.D.R.); 2 Department of Chemical Science and Technologies, University of Rome “Tor Vergata”, Via della Ricerca Scientifica 1, 00133 Rome, Italy; fabio.domenici@uniroma2.it

**Keywords:** neuro-oncology, PET/CT, brain tumors, radioligand therapy, interventional neuroradiology, meningioma, glioblastoma

## Abstract

Recurrent brain tumors—including high-grade gliomas, brain metastases, and aggressive meningiomas—continue to carry a poor prognosis, with high mortality despite therapeutic advances. The aim of this narrative review is to summarize and critically discuss the current evidence on the role of intra-arterial radioligand therapy (RLT) in the treatment of recurrent brain tumors. RLT, a targeted form of radionuclide therapy, has gained increasing attention for its potential theranostic applications in neuro-oncology. A literature search was conducted using PubMed and Scopus, including clinical studies evaluating intra-arterial radioligand delivery in central nervous system tumors. Recent research has explored intra-arterial administration of radioligands targeting somatostatin receptors and prostate-specific membrane antigen (PSMA). Somatostatin receptors are overexpressed in meningiomas, while PSMA is highly expressed in the neovasculature of glioblastomas and brain metastases; both targets can be addressed using lutetium-177 (^177^Lu)- or actinium-225 (^225^Ac)-labeled radiopharmaceuticals, traditionally delivered intravenously. Available evidence indicates that the intra-arterial route achieves markedly higher radionuclide uptake on ^68^Ga-PSMA-11 and ^68^Ga-DOTATOC PET, as well as increased absorbed doses in dosimetric models. Dosimetric analyses consistently show greater tracer accumulation compared with intravenous administration, without evidence of significant peri-procedural toxicity. Uptake in healthy brain tissue is minimal, and no relevant differences have been reported in liver or salivary gland accumulation between intra-arterial and intravenous RLT. Although based on heterogeneous and limited data, intra-arterial RLT appears to be a promising therapeutic strategy for recurrent brain tumors. Future research should focus on improving radioligand delivery beyond the blood–brain barrier and enhancing effective tumor targeting.

## 1. Introduction

Brain tumors represent a heterogeneous group of primary and secondary neoplasms, encompassing intrinsic central nervous system malignancies—such as gliomas and meningiomas—and metastatic lesions arising from a wide range of systemic cancers, many of which are associated with a poor prognosis [1,2]. Treatment of brain tumors depends on the histology, size, and location and often includes a combination of surgery, radiation therapy, and chemotherapy. Gliomas are the most common primary intra-axial brain tumor and represent 81% of primary malignant brain cancer; almost half of gliomas are glioblastomas, whose prognosis is extremely poor, with a five-years survival rate below 10% [3]. Meningiomas are the most frequently reported primary intracranial tumors; they are usually benign, slow-growing, and low-grade neoplasms in 80% of cases and have indolent growth. The remaining 20% of meningiomas—WHO grade II and III—might show aggressive or malignant behavior and carry higher risk of recurrence and mortality [4].

Brain metastases are the most common intracranial tumors and occur in up to 26% of patients with malignancies [5]. Only a few treatment options are available for progressive brain tumors. Great concerns arise when surgery and radiation therapy have failed or are not feasible due to tumor location or when the maximal acceptable dose has already been delivered to the surrounding healthy tissues by stereotactic radiation therapy [6]. At recurrence, standard of care is less defined and includes various form of pharmacological therapies, such as alkylating agents (e.g., temozolomide or lomustine), antiangiogenic treatments (e.g., bevacizumab), targeted therapies in selected molecular subtypes, and, more recently, immunotherapeutic approaches, although with overall limited and heterogeneous clinical benefit [7]. Innovative experimental therapies for glioblastoma include small-molecule-, gene-, and cell-based treatments [8]; targeted molecular therapy and experimental radiotherapy represent promising treatments for atypical and malignant meningioma [9].

Radioligand therapy (RLT) is an approach of targeted nuclear medicine treatments in which a radiolabeled molecule binds to a specific biological target—such as a receptor (e.g., somatostatin receptors in Peptide Receptor Radionuclide Therapy/PRRT) or a membrane antigen (e.g., prostate-specific membrane antigen/PSMA in prostate cancer) [10,11]. RLT represents the therapeutic component of a theranostic strategy, where a diagnostic radiopharmaceutical targeting the same molecular pathway is first used to assess expression, select patients, quantify uptake, and guide personalized dosing before delivering the therapeutic radiopharmaceutical [12,13,14].

Theranostics represents a precision-medicine strategy that integrates molecular imaging and targeted radionuclide therapy using the same biological pathway. The process begins with a diagnostic phase, typically performed with positron emission computed tomography (PET) or single-photon emission computed tomography (SPECT), in which a radiolabeled tracer targeting a specific receptor or antigen is administered to visualize its in vivo expression and distribution [15]. This imaging step confirms target presence, assesses disease burden, quantifies radiotracer uptake, and determines patient eligibility for therapy.

Once sufficient target expression is demonstrated, the corresponding therapeutic radiopharmaceutical—labeled with a cytotoxic radionuclide but using the same vector—is administered to deliver targeted radiation to tumor cells while sparing surrounding tissues. This imaging-guided, target-matched approach enables individualized treatment selection, optimization of dosing, and real-time response assessment, forming the foundation of modern theranostic practice.

RLT delivers cytotoxic radiation by combining a therapeutic radionuclide with a ligand that targets cancer cells that express a specific biomarker [16]. Within the theranostic approach to brain tumors, relevant molecular targets include somatostatin receptors (SSTRs)—commonly overexpressed in meningiomas—and prostate-specific membrane antigen (PSMA), which is highly expressed in the neovasculature of glioblastomas and brain metastases. These targets can be exploited for intravenous RLT using β^−^-emitting radionuclides such as yttrium-90 (^90^Y) and lutetium-177 (^177^Lu) or α-emitters such as actinium-225 (^225^Ac), following prior confirmation of target expression on PET or SPECT imaging.

Few reports on intravenous RLT for high-grade gliomas revealed no benefit in terms of survival, whereas intravenous RLT for meningiomas is often well tolerated and results in disease control in most cases [17].

Recently, intra-arterial (IA) delivery of somatostatin-receptor- and PSMA-targeted radiopharmaceuticals has been explored in several central nervous system tumors. Selective IA RLT involves infusing the therapeutic radioligand directly into the arterial vessels supplying the tumor, providing a concentrated local pharmacologic dose. This focused administration is expected to substantially enhance radiopharmaceutical uptake within the tumor microenvironment compared with conventional intravenous delivery, in which tracer distribution is systemic during both the diagnostic and therapeutic phases.

IA RLT bridges nuclear medicine and interventional neuroradiology and allows for a comprehensive and integrated approach to brain cancers; similar treatments are well established in patients with neuroendocrine tumors and liver metastases [18]. The purpose of this narrative review is to summarize and structurally organize the existing evidence on IA radiopharmaceutical administration in brain tumors, explicitly distinguishing diagnostic imaging, dosimetric modeling, and therapeutic studies, in order to clarify the current developmental stage of the field and delineate the prerequisites for future prospective trials.

## 2. Materials and Methods

We performed a structured literature search of the PubMed and Scopus databases on 31 October 2025 to identify clinical studies reporting IA radioligand or radiopharmaceutical administration for intracranial neoplasms. The search covered publications from 1 January 2015 through 31 October 2025. The primary search strategy used the following keywords and Boolean operators: “intraarterial” OR “intra-arterial” AND “radiopharmaceutical” AND (“meningioma” OR “glioblastoma” OR “brain tumors”). Search results were filtered for English-language and human studies. No additional databases were searched. Studies were selected according to pre-specified inclusion and exclusion criteria.

Inclusion criteria were clinical studies (prospective or retrospective), clinical trials, case series, and case reports reporting IA administration of radiopharmaceuticals or radioligands for primary or metastatic brain tumors. Exclusion criteria included preclinical studies (in vitro or animal models), narrative or systematic reviews, editorials, commentaries, and conference abstracts for which a full manuscript was not available. Studies not published in English were also excluded.

**Figure 1:**
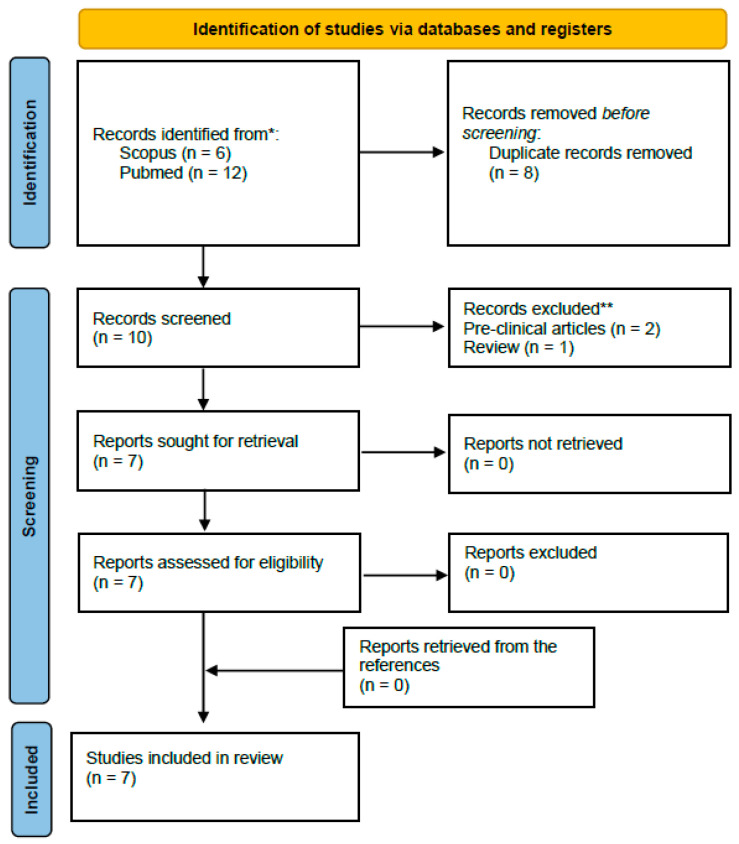
PRISMA flowchart indicating the selection process of the included studies.

Study screening and selection were performed independently by two reviewers (F.S., M.T.), using a two-step process (title and abstract screening followed by full-text review). Disagreements were resolved by consensus; when consensus could not be reached, one of the senior authors (L.F.) served as an adjudicator. Data extracted from the included studies comprised study design, number of patients, tumor type, radiopharmaceutical used, procedural characteristics, safety outcomes, and efficacy and/or imaging results. A PRISMA-style flow diagram summarizing the numbers of records identified, screened, excluded, and included is provided in Figure 1.

## 3. Results

A total of seven reports addressing IA approaches in brain tumors were identified. According to the predefined classification, three distinct lines of evidence emerged: (1) IA diagnostic imaging (*n* = 1 studies), (2) IA administration for RLT enrollment and dosimetric modeling (*n* = 1), and (3) therapeutic applications (*n* = 5), as detailed in the subsequent sub-sections.

A summary of the main findings on IA-based theranostic approaches for brain tumors is provided in Table 1.

### 3.1. Intra-Arterial Diagnostic Imaging

Intracranial hemangiopericytomas are rare and aggressive tumors characterized by high rates of local recurrence [26]. Similar to meningiomas, hemangiopericytomas may exhibit high expression of SSTRs [27], making them potential candidates for evaluation with IA tracers targeting SSTRs. Veldhuijzen van Zanten et al. [20] reported an illustrative imaging case involving a 40-year-old woman with an intracranial hemangiopericytoma evaluated using ^68^Ga-DOTATATE PET/CT. Following standard intravenous administration, the lesion demonstrated only moderate radiotracer uptake, corresponding to a Krenning score of 2. The investigators subsequently performed a selective IA injection of ^68^Ga-DOTATATE via the posterior cerebral artery and repeated PET imaging. This approach resulted in a marked increase in lesional uptake, with tumor activity exceeding that of the liver, consistent with a Krenning score of 3. Quantitatively, the mean standardized uptake value (SUV_mean) increased from 8.4 to 21.0, while the maximum standardized uptake value (SUV_max) rose from 15.8 to 36.0. Based on these findings, the authors suggested that selective arterial delivery of SSTR-targeting radiotracers may enhance tumor uptake sufficiently to support the feasibility of PRRT in selected central nervous system tumors.

### 3.2. Intra-Arterial Administration for RLT Enrollment and Dosimetric Modeling

The first proof of concept of IA PSMA-RLT for brain tumors by Pruis et al. [21] proved higher tumor uptake of PSMA-based compound (^68^Ga-PSMA-11) after superselective IA administration compared to intravenous administration. The endovascular approach was performed in 10 patients suffering from IDH wild-type glioblastoma (*n* = 4), oligodendroglioma (*n* = 1), and metastatic non-small-cell lung cancer (*n* = 4) or breast cancer (*n* = 1). Arterial access was gained through a trans-femoral approach; the dominant feeding vessels were identified with digital subtraction angiography and cone-beam computed tomography, and subsequent super-selective catheterization was performed. Most feeding vessels were distal branches of cerebral or cerebellar arteries; branches from the middle meningeal arteries were involved in 30% of cases. Patients received a median dose of 82 MBq after intra-arterial administration with a 5-day median interval after intravenous administration and underwent PET/MRI at 90 min, 165 min, and 240 min after injection; endovascular procedures were well tolerated (except in one case, where a transient stroke-like syndrome was reported, probably due to vascular spasm or contrast-induced encephalopathy), and all patients accomplished the study. A median 15-fold higher uptake at the tumor site after IA injection with respect to intravenous administration was confirmed after semi-quantitative analysis. Of note is the fact that IA administration also allowed for dosimetric modeling for ^177^Lu- or actinium-based RLT (^177^Lu-PSMA- or ^225^Ac-PSMA-based RLT), given its potential to deliver high and focused radiation doses in the tumoral microenvironment.

PSMA is highly expressed on high-grade gliomas vasculature and, to a lesser extent, on gliomas and metastasis cells membrane. Radionuclide uptake was deemed negligible in surrounding healthy brain tissue and comparable to intravenous administration [28]; in contrast, salivary glands uptake was moderate-to-high for both administration routes with no significant differences. Of note is the fact that estimation of radionuclide uptake in the salivary glands is mandatory to qualify patients for PSMA-targeted RLT and might potentially limit the treatment, according to the joint Society of Nuclear Medicine and Molecular Imaging/European Association of Nuclear Medicine guidelines for ^177^Lu-labeled RLT [29]: if the tumor/salivary gland SUV ratio is <0.5, patients should be excluded from receiving ^177^Lu-PSMA-RLT. As mentioned above for meningiomas, the same inclusion criteria for intravenous RLT were applied for patient selection; intra-arterial administration of ^68^Ga-PSMA-11 qualified the totality of patients for intra-arterial RLT. Again, given the small volume of PSMA-expressing cells compared to other clinical scenarios (i.e., metastatic castration-resistant prostate cancer with high tumor burden), the “sink effect” is unlikely to occur, and the biodistribution beyond the brain tissue of PSMA-based compounds injected intra-arterially is comparable to intravenous administration.

### 3.3. Therapeutic Applications

Meningiomas are considered refractory when local control cannot be achieved with standard treatments such as surgery, radiotherapy, or chemotherapy. Overexpression of somatostatin receptor subtype 2a (SSTR2a) is common in meningiomas [30] and, as in neuroendocrine tumors, enables sensitive PET/SPECT imaging for diagnosis and selection for PRRT [31]. SSTR2a expression also supports intraoperative radioguided surgery, improving lesion localization and completeness of resection [32]. Assessment of SSTR2a by PET imaging with radiolabeled tracers such as ^68^Ga-DOTATOC, ^68^Ga-DOTATATE, or ^68^Ga-DOTANOC is typically required to determine suitability for intravenous RLT [33].

**Figure 2:**
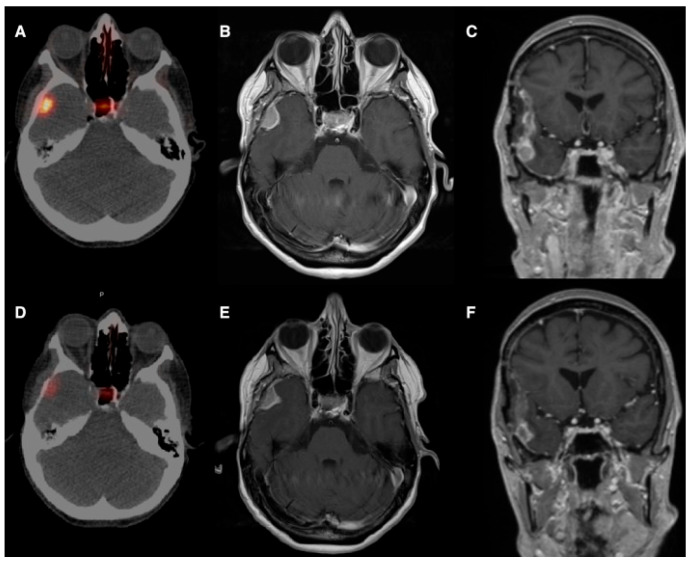
(**A**). Baseline ^68^Ga-DOTATOC PET/CT shows high SSTR expression in the right-temporal meningioma and normal pituitary uptake. (**B**,**C**). Gadolinium-enhanced T1 MRI shows a uniformly enhancing solid lesion. (**D**). Post-treatment gallium-68-DOTATOC PET/CT shows a 79% decrease in SSTR2 expression. (**E**,**F**). Post-treatment T1 MRI shows a partial response with a 38% volume and 24% diameter reduction, central necrosis, and reduced enhancement in the prior resection cavity. Reprinted from [19] under a creative commons attribution 4.0 international license (http://creativecommons.org/licenses/by/4.0/, accessed on 31 October 2025). No changes were made.

**Figure 3:**
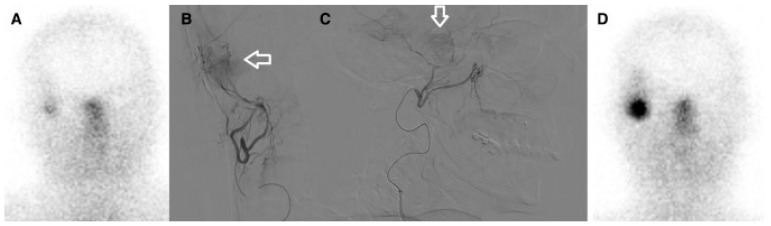
(**A**) Post-treatment ^177^Lu-HA-DOTATATE scan after intravenous administration shows only faint radiotracer uptake in the meningioma. (**B**,**C**). Anterior and lateral digital subtraction angiography during selective injection in the right external carotid artery (middle meningeal branch), just proximal to the parotid artery origin, demonstrates a clear tumor blush (white arrows). This position was selected because an additional feeding branch arose from the parotid artery. (**D**). After intra-arterial administration, the post-treatment ^177^Lu-HA-DOTATATE scan shows a marked increase in radiotracer uptake, with quantification demonstrating an approximately 11-fold enhancement. Reprinted from [19] under a creative commons attribution 4.0 international license (http://creativecommons.org/licenses/by/4.0/, accessed on 31 October 2025). No changes were made.

The first experience by Braat et al. [19] provided the rationale for selective vascular delivery in meningioma. They reported the case of a 54-year-old woman with a recurrent right-temporal WHO grade II meningioma, refractory to three surgical resections and seven courses of radiotherapy, presenting with frequent focal seizures and recurrent status epilepticus. As the tumor was deemed unresectable and further external radiotherapy unsafe, ^68^Ga-DOTATOC PET/CT demonstrated high SSTR2 expression, prompting treatment with PRRT. An initial intravenous administration of 7.4 GBq ^177^Lu-DOTATATE resulted in low tumor uptake and an estimated absorbed dose of approximately 4.6 Gy. Subsequently, selective IA administration via the right external carotid artery led to an 11-fold increase in tumor uptake, achieving an estimated absorbed dose of 51 Gy per cycle. After four PRRT cycles (one intravenous and three IA; cumulative activity 29.6 GBq), the estimated total tumor absorbed dose reached 154 Gy. Follow-up PET/CT and MRI demonstrated a marked reduction in SSTR2 expression, central tumor necrosis, and a partial radiological response. Clinically, severe seizures resolved completely, while focal sensory seizures decreased from daily episodes to 3–4 per week, with stable control and sustained partial response at 10-month follow-up and no relevant treatment-related toxicity (Figure 2 and Figure 3).

Vonken et al. [22] presented a retrospective intrapatient comparison (*n* = 4 IA-treated patients, selected from 7 referred) showing that selective IA administration of ^177^Lu-HA-DOTATATE after an initial intravenous cycle produces a large and consistent increase in intratumoral tracer accumulation (median planar target-to-background ratio increased from 1.7 to 3.7; SPECT/CT ratio increased from 15.0 to 59.8). The IA procedure was technically successful in 100% of attempted cases and produced no angiography-related complications. Clinically, three WHO grade 2 patients completed four cycles (one partial response, two stable disease), while a WHO grade 3 patient progressed and died; follow-up median was 1.7 years. Toxicity was limited and acceptable (one isolated grade 3 leukopenia). The authors concluded that IA PRRT can be feasible and safe and yields a favorable tumor-to-background delivery profile, warranting prospective investigation.

In the paper by Puranik et al. [23], the authors reported the initial experience of a tertiary neuro-oncology center using peptide PRRT with ^177^Lu-DOTATATE in patients with treatment-refractory, progressive meningioma, with a specific focus on the added value of IA RLT. Eight patients with recurrent or progressive meningioma (WHO grades I–III), previously treated with surgery, radiotherapy, and in some cases chemotherapy, were included. All patients demonstrated significant SSTR-expression on ^68^Ga-DOTANOC PET/CT and were selected for PRRT following multidisciplinary tumor board discussion. PRRT was administered in cycles of 7.4 GBq (200 mCi) ^177^Lu-DOTATATE, with all patients receiving an intravenous cycle to ensure systemic disease coverage. In four patients, subsequent cycles were delivered via a selective IA approach after digital subtraction angiography identified tumor-feeding vessels. IA administration was technically successful in all cases, without peri-procedural complications, highlighting the feasibility of this interventional radionuclide therapy when performed in a specialized setting. Dosimetric analysis demonstrated that IA administration resulted in significantly higher tumor absorbed dose and longer residence time compared with IV delivery, while simultaneously reducing radiation exposure to organs at risk such as kidneys, liver, and spleen. Mean tumor absorbed dose increased from 2.86 Gy with intravenous administration to 3.62 Gy with IA administration, with the absorbed dose per unit activity nearly doubling (0.82 vs. 1.72 Gy/GBq). These findings confirmed a clear pharmacokinetic advantage of first-pass IA delivery, enabling more efficient tumor targeting and improved therapeutic index. From a clinical efficacy standpoint, early post-therapy MRI assessment using RANO criteria showed stable disease or partial response in the majority of patients after two PRRT cycles. Median time to progression was 8.9 months, with better disease control observed in WHO grade I–II meningiomas compared with grade III tumors, which demonstrated poor outcomes despite therapy. Response assessed on PET correlated well with anatomic response on MRI, supporting the role of functional imaging in treatment assessment. Importantly, PRRT was associated with symptomatic improvement, even in the absence of marked tumor shrinkage. Treatment was well tolerated, with no significant PRRT-related or angiography-related toxicities and no grade ≥3 non-hematologic adverse events.

Amerein et al. [24] reported a single-center retrospective experience with IA PRRT using ^177^Lu-HA-DOTATATE in patients with progressive, advanced meningioma who were not candidates for further surgery or radiotherapy. Thirteen patients with SSTR–positive disease on pre-therapeutic ^68^Ga-DOTATOC PET/CT underwent selective IA administration of the radioligand. Each treatment cycle delivered approximately 6.0–7.7 GBq (mean about 7.4 GBq) of ^177^Lu-HA-DOTATATE, with up to four cycles per patient and a mean cumulative activity of about 25.7 GBq; standard renal protection with lysine and arginine amino acid infusion and antiemetic prophylaxis was administered according to established PRRT protocols. The treatment was generally well tolerated, with predominantly transient hematologic toxicity, particularly lymphocytopenia, infrequent grade 3 or higher adverse events, and no clear evidence of chronic PRRT-related nephrotoxicity; complications related to angiography or catheterization were rare. Radiologic evaluation based on volumetric RANO criteria showed a high rate of disease control, with complete or partial response or stable disease observed in the majority of evaluable patients, and this was paralleled by stabilization or improvement of clinical symptoms in most cases; the median progression-free survival was 18 months and varied according to WHO grade. Although arterial embolization was performed in some patients during later treatment cycles and may have influenced outcomes, the study concluded that IA delivery of ^177^Lu-based PRRT was feasible and showed promising therapeutic activity in refractory meningioma, supporting further prospective investigations incorporating patient-specific dosimetry to better define its efficacy and safety relative to intravenous PRRT.

A recently published retrospective multicenter cohort study evaluated the efficacy and safety of IA administration of ^177^Lu-DOTATATE monotherapy in patients with treatment-refractory meningioma who were no longer eligible for surgery or external beam radiotherapy [25]. Seventeen patients with SSTR-positive disease on ^68^Ga-DOTATOC PET/CT received selective IA PRRT. The intended treatment regimen consisted of up to four cycles of approximately 7.4 GBq per cycle, with a median of three IA cycles administered and a median cumulative activity of 28.8 GBq. With a median follow-up of 36 months, the study reported a 6-month progression-free survival of 65% and an overall survival of 82%, with an objective response rate of 24% and a disease control rate of 53% according to RANO criteria, outcomes that compared favorably with historical benchmarks of intravenous PRRT. Treatment was generally well tolerated, with limited grade 3 toxicity consisting mainly of anemia, rare treatment discontinuation due to radionecrosis or SMART syndrome likely related to prior radiotherapy, and a single angiography-related peripheral embolic complication.

### 3.4. Radiotracers in Malignant Brain Tumors, Implications for Radioligand Therapy

Over the past few years, a considerable number of studies have been published regarding the use of new PET radiotracers in the fields of gliomas. The already mentioned PSMA and the fibroblast activation protein (FAP) can be both targeted with ^68^Ga-based compound for diagnostic purposes and have potential role in the radiation therapy planning.

PSMA is not only expressed on prostate cancer cells, but also in the microenvironment of a large variety of solid organ tumors including primary and secondary brain tumors, especially in the microvasculature of high-grade gliomas (HGGs). FAP is expressed by cancer cells and cancer-associated fibroblasts in glioblastomas, where it promotes glioma local invasion, and in epithelial cancers. These statements make both PSMA and FAP potential targets for PSMA- and FAP-targeted PET imaging radiotracers and allow for potential RLT and theranostic opportunities.

^68^Ga-labeled PSMA-targeted compounds have shown promising results in detection of HGG, given the 100% concordance between MRI and PET in a series of 49 lesions by Kumar et al. [34]; the inherently high lesion-to-background ratio, along with the lack of tracer uptake in radiation necrosis, allows for reliable detection of glioma recurrence after radiotherapy. Furthermore, a comparison between PET/CT with ^68^Ga-PSMA-11 and ^18^F-fluorodeoxyglucose (^18^FDG) demonstrated a higher tumor-to-background ratio and superior detection rate of recurrent gliomas with the PSMA-based tracer in the preliminary analysis by Sasikumar et al. In addition, ^68^Ga-PSMA-11 SUVmax correlates with Ki-67 expression and higher tumor grades on histopathology [35].

The use of PSMA-based compounds in diagnostic nuclear medicine imaging is thus promising, but their theranostic application for intravenous radioligand therapy seems questionable. Few case series based on a single-center experience report conflicting results, notably in terms of intratumoral radiation distribution and delivered therapeutical dose [36]; further, the lack of randomized trials, guidelines, and standardization among centers makes it challenging to obtain solid evidence-based results on this topic.

At the same time, many fibroblast activation protein inhibitors (FAPI) have been developed to bind FAP, labeled with ^68^Ga or ^18^F [37]. Similarly to PSMA-based compounds, FAP-based radiotracers show high accumulation in primary glial tumors and exhibit high a tumor-to-background ratio notably in HGG (grade 3 and 4) as well as in IDH wild-type glioblastomas [38]. These findings are supported by the significant correlation of immunohistochemical FAP with SUV mean and SUV peak of ^68^Ga-FAPI-46 [39] in glioblastomas and gliosarcomas. For tumors of epithelial origin, the lack of accumulation in the healthy brain tissue makes FAPI-based imaging superior to ^18^FDG in the detection of brain metastases [40].

## 4. Discussion

Preliminary experience on IA RLT for refractory meningiomas, although limited and inferred by few case reports and case series, paves the way for further research, as results seem satisfactory in terms of disease control and rates of adverse events. Grade 3 meningiomas show rapid growth, might invade the brain tissue and their outcome is a priori unfavorable compared to grade 1 and 2. It should be noted that, according to the current European Association of Neuro-Oncology (EANO) guidelines, intravenous RLT is yet considered an experimental therapy to be reserved for grade 3 meningiomas; IA-based treatments are not even mentioned in the guidelines and should be considered even more than experimental. These statements appear inconsistent with the limited clinical evidence available, as treatment with both intravenous and IA RLT might be safely performed in all WHO grade meningiomas, and patients with the most beneficial effects seem to be those affected by grade 1 and 2 meningiomas, given their less aggressive nature compared to grade 3. More consistent results are needed from a larger sample size in randomized trials, with standardized patient selection (both for tumor grade and previous treatment options) and individual dosimetry.

IA RLT is potentially feasible also in HGG and secondary brain tumors, based on the proof of concept by Pruis et al. [21]. However, careful thoughts are needed, given the higher expression of PSMA on tumor microvasculature rather than on tumoral cell membrane: to reach the tumor, adequate tissue penetration is required. RLT with β-emitters seems mandatory to reach tumor DNA effectively; in contrast, α-emitters like ^225^Ac have high ionizing energy but short penetration in tissues. Compared to other β-emitters such as ^177^Lu, ^90^Y has higher energy, longer tissue penetration, and higher chance to reach tumor cells and their DNA. In addition, in the case of ^90^Y, the therapeutic relevance of the crossfire effect should be considered, particularly in tumors in which the molecular target is predominantly or exclusively expressed on the tumor-associated microvasculature rather than on the neoplastic cells themselves. When radiopharmaceuticals are labeled with sufficiently long-range particle emitters, such as ^90^Y (i.e., average tissue penetration range: 2.5 mm, maximum range of up to 11 mm), radiation emitted from targeted endothelial cells can extend beyond the site of binding and deliver a cytotoxic dose to adjacent tumor cells. This mechanism may partially overcome limitations related to heterogeneous or low target expression within the tumor mass and supports the rationale for targeting the tumor microenvironment as an indirect yet effective therapeutic strategy. Consequently, the crossfire effect may expand the spectrum of tumors amenable to targeted radionuclide therapy, even in cases where direct tumor cell targeting is suboptimal [15].

Of note is the fact that, according to Brighi et al. [41], the PSMA-based compound ^68^Ga-PSMA-617 exhibits lower nonspecific affinity to the kidneys compared to ^68^Ga-PSMA-11, used in the proof of concept by Pruis and coworkers [21]; in the absence of a proper renal protection, the likelihood of nephrotoxicity is a major concern because kidneys represent the possible dose-limiting organ for RLT [42]. ^68^Ga-PSMA-617 accumulates in HGGs and beyond tumors margin, in zones of early neoangiogenesis without gadolinium enhancement in MRI where the blood–brain barrier is not yet disrupted; thanks to its features, it might be considered a potential and solid tool for theranostic applications. As ^68^Ga-PSMA-11 accumulates in areas of blood–brain barrier disruption, its efficacy in RLT might be limited and it might lack efficacy in the peritumoral environment of infiltrating tumor cells beyond the contrast-enhancing margins.

Further methods have been investigated in vitro to boost radioligand delivery in the tumoral tissue, such as biocompatible polyvinyl alcohol (PVA) microbubbles labeled with ^90^Y [43]; these particles can be functionalized with a specific endothelial target overexpressed on tumor-invasive endothelia and cells and have the potential advantage to open the blood–brain barrier when stimulated by ultrasound [44]. Active targeting of PVA microbubbles was tested on human umbilical vein endothelial cell (HUVEC) lines expressing integrins αvβ3, highly represented on HGGs and tumor-invasive endothelia; derivatization of microbubbles with a Cyclo(Arg-Gly-Asp-D-Phe-Lys) ligand peptide enabled specific interaction with the integrin receptors overexpressed on the endothelium of the HUVEC cell model for the proof of concept of a new radioembolization platform.

Despite encouraging early reports of enhanced tumor uptake with super-selective IA administration, the existing evidence base remains limited to small, heterogeneous case series and single-center feasibility studies. To translate this approach into clinical practice, prospective phase I/II trials are required that combine (1) standardized patient selection criteria (histology, prior treatments, and quantitative PET thresholds), (2) harmonized imaging and dosimetric protocols to permit inter-study comparison, and (3) predefined safety endpoints focused on neurological and systemic toxicity. Incorporating advanced delivery techniques—for example, transient blood–brain barrier modulation via focused ultrasound, catheter-directed microbubble platforms, or nanoparticle carriers—could increase intratumoral penetration while keeping off-target exposure minimal. Parallel translational work should prioritize multi-regional tumor sampling and paired PET-guided biopsies to correlate imaging uptake with target expression and microenvironmental features (e.g., vascular density, SSTR/FAP/PSMA expression). Finally, given the differing tissue penetration profiles of β-emitters and α-emitters, early trials should explicitly match radionuclide choice to expected target geometry and required range of energy deposition; adaptive dosimetry and individualized administration schedules will likely be critical to maximize efficacy while maintaining organ-at-risk constraints.

Although well established in selective internal radiation therapy (SIRT) for liver cancer [45], standardization is lacking in the neuro-oncological field despite several reports on IA infusion of chemotherapeutic agents and monoclonal antibody. Arteriography and selective catheterization preliminary to drug delivery are safely performed on a routine basis in high-volume neurovascular centers, as shown by the reported experience in brain tumors—notably in meningiomas embolization—as well as in the daily practice in the endovascular treatment of stroke, aneurysms, and vascular malformation. Endovascular embolization of meningiomas or paragangliomas is part of the skills of interventional neuroradiologist, but most of them are not used to treat malignancies; thanks to the expertise in vessel navigation and management of complex clinical scenarios, the opportunity to treat brain cancer with endovascular means seems cutting-edge.

The current evidence base for IA RLT in brain tumors remains preliminary and is subject to several important limitations that affect the robustness and generalizability of the conclusions. Notably, at the time of writing, no registered ongoing or pending clinical trials specifically investigating IA radioligand therapy or PRRT for brain tumors were identified in ClinicalTrials.gov (accessed 31 October 2025), underscoring the exploratory and emerging nature of this therapeutic approach. This lack of registered clinical trials may be explained by several factors.

First, publication bias is likely: reports to date are predominantly single-center case reports and series that tend to emphasize positive imaging “boost” results, and negative or neutral experiences may be under-reported. Second, very small sample sizes and single-center experiences dominate the field; most series include fewer than 15 treated patients, and several reports are single-patient case reports, which prevents robust estimation of efficacy and uncommon toxicities. Third, heterogeneity in eligibility criteria and imaging thresholds (different SUV/ratio thresholds, differing reference organs, and visual scores) limits comparability across centers. Fourth, short imaging follow-up in diagnostic IA studies (minutes to hours) prevents assessment of sustained tracer retention or delayed toxicity and complicates translation to therapeutic dosing. Fifth, procedural and technical variables (catheter tip position, infusion rate, administered activity, and timing of imaging) were often incompletely reported, further increasing heterogeneity. In order to facilitate reproducibility, we therefore recommend that future reports adopt the minimum reporting items presented in Table 2.

Finally, statements relating to the absence of significant toxicity must be interpreted cautiously: available safety data are limited in sample size and follow-up and cannot exclude rare neurological or systemic events. These limitations underscore the need for prospective, multicenter phase I/II trials with harmonized selection criteria, standardized imaging/dosimetry protocols, prespecified safety endpoints, and adequate follow-up.

## 5. Conclusions

Preliminary clinical experience with IA RLT suggests its potential as a targeted, theranostic approach for selected brain tumors, with indications of increased IA delivery compared with systemic administration. To further explore its clinical applicability, coordinated multicenter efforts are warranted to (1) standardize eligibility criteria and dosimetry, (2) assess safety and preliminary efficacy in phase I/II trials, and (3) incorporate translational correlative studies to support the validation of imaging biomarkers. Close collaboration between nuclear medicine, neurointerventional teams, and medical physics will be important to carefully advance intra-arterial RLT toward routine oncologic practice.

## Figures and Tables

**Table 1:** Intra-arterial theranostic approaches in brain tumors.

Study (Year)/Country	Study Type	Tumor Type; *n*	Tracer/Therapeutic Agent	Key Quantitative Uptake Metrics (IA vs. IV)	Median Follow-Up	Main Outcomes	Notable AEs
Braat et al., 2019 [19]/The Netherlands	Case report	Recurrent right-temporal meningioma (WHO II); *n* = 1 (54 y, F)	^177^Lu-DOTATATE (IV then IA)	IV absorbed dose ≈ 4.6 Gy vs. IA ≈ 51 Gy per cycle; ~11× uptake increase	10 months (single patient)	Partial radiologic response (38% volume reduction), 79% decrease in SSTR2 expression on PET; clinical seizure control	No relevant treatment-related toxicity reported
Veldhuijzen Van Zanten et al., 2021 [20]/The Netherlands	Case report/ 40-year-old	Intracranial hemangiopericytoma; *n* = 1 (40, F)	^68^Ga-DOTATATE (arterial injection)	Doubling of maximum SUV (IA vs. IV)	Not reported (early clinical deterioration; no IA therapy performed)	Selective IA administration increased tumor uptake (SUV_mean: 8.4 → 21.0; SUV_max: 15.8 → 36.0), suggesting potential feasibility of PRRT	Not applicable/not reported
Pruis et al., 2024 [21]/The Netherlands	Single-center, open-label, non-randomized prospective imaging study/	Glioblastoma (IDH-wt) *n* = 4; oligodendroglioma *n* = 1; brain mets (NSCLC *n* = 4, breast *n* = 1); total *n* = 10 (8 M)	^68^Ga-PSMA-11 (IA vs. IV diagnostic imaging; median IA activity ≈ 82 MBq)	Median ~15× higher tumor uptake after IA vs. IV (semi-quantitative analysis); imaging acquired 90, 165, 240 min post-injection	Imaging-only study: short-term imaging up to 240 min p.i. (no long-term median follow-up reported)	IA increases tumor uptake enabling dosimetric modeling for ^177^Lu- or ^225^Ac-based RLT; all patients qualified for IA RLT based on IA imaging	One transient stroke-like syndrome (probable vascular spasm/contrast encephalopathy); otherwise well tolerated
Vonken et al., 2022 [22]/The Netherlands	Retrospective intrapatient comparison (selected patients)	Salvage meningioma patients; *n* = 4 IA-treated (selected from 7 referred), age: 44–66 y	^177^Lu-HA-DOTATATE (IV then IA)	Planar target-to-background ratio median: 1.7 (IV) → 3.7 (IA); SPECT/CT ratio: 15.0 (IV) → 59.8 (IA)	Median follow-up 1.7 y	IA PRRT feasible and safe; 3 patients completed 4 cycles (1 PR, 2 SD); 1 WHO grade 3 patient progressed and died	One isolated grade 3 leukopenia; no angiography-related complications reported
Puranik et al., 2024 [23]/India	Single-center initial experience/case series	Treatment-refractory progressive meningioma (WHO I–III); *n* = 8 (5 M), median age: median age–52.3 t	^177^Lu-DOTATATE PRRT (IV cycle for systemic coverage; subsequent IA cycles in 4 patients); 7.4 GBq per cycle	Mean tumor absorbed dose: 2.86 Gy (IV) → 3.62 Gy (IA); absorbed dose per unit activity: 0.82 Gy/GBq (IV) → 1.72 Gy/GBq (IA)	Median time to progression 8.9 months (study reports this efficacy timeframe)	Majority with stable disease or partial response after two cycles; metabolic PET response correlated with MRI; symptomatic improvement reported	No significant PRRT-related or angiography-related toxicities; no grade ≥3 non-hematologic AEs reported
Amerein et al., 2024 [24]/Germany	Single-center retrospective series	Progressive, advanced meningioma (SSTR-positive); *n* = 13 (8 F); mean age: 65 ± 13 y	^177^Lu-HA-DOTATATE IA; per-cycle activity ≈ 6.0–7.7 GBq (mean ≈ 7.4 GBq); up to 4 cycles; mean cumulative ≈ 25.7 GBq	Angiography was successful in all cases (100%). A mean activity of 7.4 GBq per cycle administered without dose reductions, resulting in a mean cumulative activity of 25.7 GBq.	Median progression-free survival reported ≈ 18 months	High rate of disease control (CR/PR/SD in majority); clinical symptom stabilization/improvement; IA PRRT feasible with promising activity	Predominantly transient hematologic toxicity (notably lymphocytopenia); infrequent grade ≥3 AEs; no clear chronic nephrotoxicity; rare angiography-related complications
El Ghalbouni et al. [25]/The Netherlands	Retrospective multicenter cohort	Treatment-refractory meningioma (WHO 1–3); *n* = 17 (11 M), median age: 64 y	^177^Lu-DOTATATE monotherapy (selective IA administration); median cycles ≈ 3; median cumulative activity ≈ 28.8 GBq	Study emphasizes IA increased tumor absorbed dose by exploiting first-pass arterial delivery	Median follow-up 36 months	6-month PFS 65%; OS 82%; objective response rate 24%; disease control rate 53% (RANO criteria); favorable vs. historical IV benchmarks	Limited grade 3 toxicity (mainly anemia); rare radionecrosis or SMART syndrome (likely related to prior radiotherapy); one angiography-related peripheral embolic complication

F: female; M: male; y: years; WHO: World Health Organization; IV: intravenous; IA: intra-arterial; Gy: Gray; SSTR: somatostatin receptor; SUV: standardized uptake value, PSMA: prostate specific membrane antigen; PET: positron emission tomography; PR: partial response; SD: stable disease; PRRT: peptide radionuclide receptor therapy.

**Table 2:** Recommended reporting items for future intra-arterial RLT studies.

Domain	Items to Report
**Patient selection**	Histology (WHO grade), prior treatments, performance status, PET/SPECT selection thresholds (SUV, ratios), renal function
**Radiopharmaceutical**	Tracer name, radionuclide (e.g., ^177^Lu, ^225^Ac, 90Y), specific activity, mass dose
**Administration technique**	Access route (femoral/radial), catheter type, super-selective vessel(s) catheterized, angiographic mapping images, infusion rate, total activity administered
**Imaging and dosimetry**	Pre-IA and post-IA PET protocol (timing, scanner), quantitative metrics (SUVmax, SUVmean, tumor/background ratios), absorbed dose estimates to tumor and organs at risk
**Safety and AEs**	Peri-procedural complications (neurological, vascular), systemic toxicity (renal, salivary gland), grade per CTCAE, time to onset
**Outcomes and follow-up**	Radiologic response criteria (RANO or RECIST as applicable), clinical symptom changes, PFS/OS if available, planned follow-up schedule
**Translational correlates**	If available, paired biopsy data, immunohistochemistry (PSMA, SSTR2a, FAP), microregional uptake correlation

WHO: World Health Organization; SUV: standardized uptake value; CTCAE: Common Terminology Criteria for Adverse Events; RANO: Response Assessment in Neuro-Oncology; PFS: progression-free survival; OS: overall survival.

## Data Availability

No new data were created or analyzed in this study.

## Abbreviations

The following abbreviations are used in this manuscript:

RLT	Radioligand therapy
WHO	World Health Organization
SSTR	Somatostatin receptor subtype
PET	Positron emission tomography
CT	Computed tomography
MRI	Magnetic resonance imaging
SUV	Standardized uptake value
PSMA	Prostate-specific membrane antigen
FAP	Fibroblast activation protein
FAPI	Fibroblast activation protein inhibitors
PVA	Polyvinyl alcohol
BBB	Blood–brain barrier
SD	Stable disease
PD	Progression disease
